# Immune drivers of venous thrombosis: orchestrating initiation, formation, and resolution within the hypoxic microenvironment

**DOI:** 10.3389/fimmu.2026.1801442

**Published:** 2026-06-10

**Authors:** Jiaojiao Yin, Enpeng Zhu, Xiaojing Zheng, Xiaoqin Ha, Bing Li

**Affiliations:** 1 Department of Clinical Laboratory, Gansu Provincial Maternity and Child-care Hospital, Lanzhou, Gansu, China; 2 Department of Clinical Laboratory, 940th Hospital of Chinese People’s Liberation Army Joint Support Force, Lanzhou, Gansu, China; 3 School of Basic Medical Sciences, Gansu University of Chinese Medicine, Lanzhou, Gansu, China

**Keywords:** endothelial cell, hypoxic microenvironment, macrophage, monocyte, neutrophil, venous thrombosis

## Abstract

Venous thromboembolism (VTE) is a prevalent and life-threatening condition whose pathogenesis extends beyond a mere coagulation disorder to encompass a dynamic interplay with the immune system, a concept known as thromboinflammation. Hypoxia, a critical but often overlooked component of the venous microenvironment, serves as a master regulator that orchestrates immune cell responses throughout the thrombotic cascade—from initiation to resolution. This review synthesizes current evidence to establish an integrated framework linking the hypoxic microenvironment to immune-mediated venous thrombosis. We detail how hypoxia, arising from external exposure, pathological stasis, or intracellular metabolic reprogramming (pseudohypoxia), reprograms the function of key cellular players. This includes endothelial cell activation with glycocalyx shedding and adhesive molecule expression, neutrophil extracellular traps (NETs) formation, and monocyte recruitment. Collectively, these hypoxia-driven events create a prothrombotic milieu. Conversely, during the resolution phase, hypoxia-inducible factor (HIF)-1α signaling promotes angiogenesis, while hypoxia-induced metabolic shifts—particularly lactate accumulation—appear to influence macrophage polarization towards a pro-resolving phenotype. By elucidating the central role of immune cells as effectors of hypoxic signaling, this review highlights the hypoxic-immune axis as a pivotal pathway in venous thrombosis and points to potential immunomodulatory strategies for future therapeutics.

## 1 Introduction

Venous thromboembolism (VTE), which consists of deep vein thrombosis (DVT) and pulmonary embolism (PE), is the third most common cardiovascular disorder, affecting up to 5% of the population (1). Approximately one-third of patients with DVT develop postthrombotic syndrome (PTS), a chronic condition characterized by persistent limb pain, swelling, redness, and ulcers, which carries a significant health and economic burden and is associated with a reduced quality of life (2). VTE incidence is almost 3‰ in the normal population but significantly increased during the COVID-19 pandemic, varying between 14.1% and 31% in hospitalized populations. Patients admitted to the intensive care unit (ICU) have a 2- to 3-fold greater incidence of VTE than those who do not need intensive care (3).

Hypoxia is considered an overlooked trigger for venous thrombosis (VT) (4). One contributing factor is venous valves (5, 6), which play a major role in helping with blood circulation in the legs and are also areas where stasis and subsequent hypoxia may occur (7). This valvular sinus stasis increases hematocrit levels, constituting a potentially hypercoagulable microenvironment. The increased coagulation, venous stasis and endothelial injury that may be induced by hypoxia and/or inflammatory mediators contribute to Virchow’s triad of VT (8). The other contributing factor comes from studies of high-altitude exposure, which perturbs the molecules associated with vascular integrity and contributes to the early onset of VT (9). Genome-wide expression analyses have suggested that hypoxia-induced hypercoagulation, characterized by the upregulation of hemostasis and platelet activation genes, contributes to VT (10). Hypoxia serves as an independent stimulus for thrombosis and is also a consequence of thrombus formation; in turn, the hypoxic milieu after thrombosis impacts the resolution of the thrombus (11–13). In this review, we will detail the relationship between hypoxia and VT, including the role of hypoxia in the initiation, formation and resolution of VT, thereby providing strategies for the prevention and treatment of VT.

## 2 The natural evolution of VT

**Figure 1:**
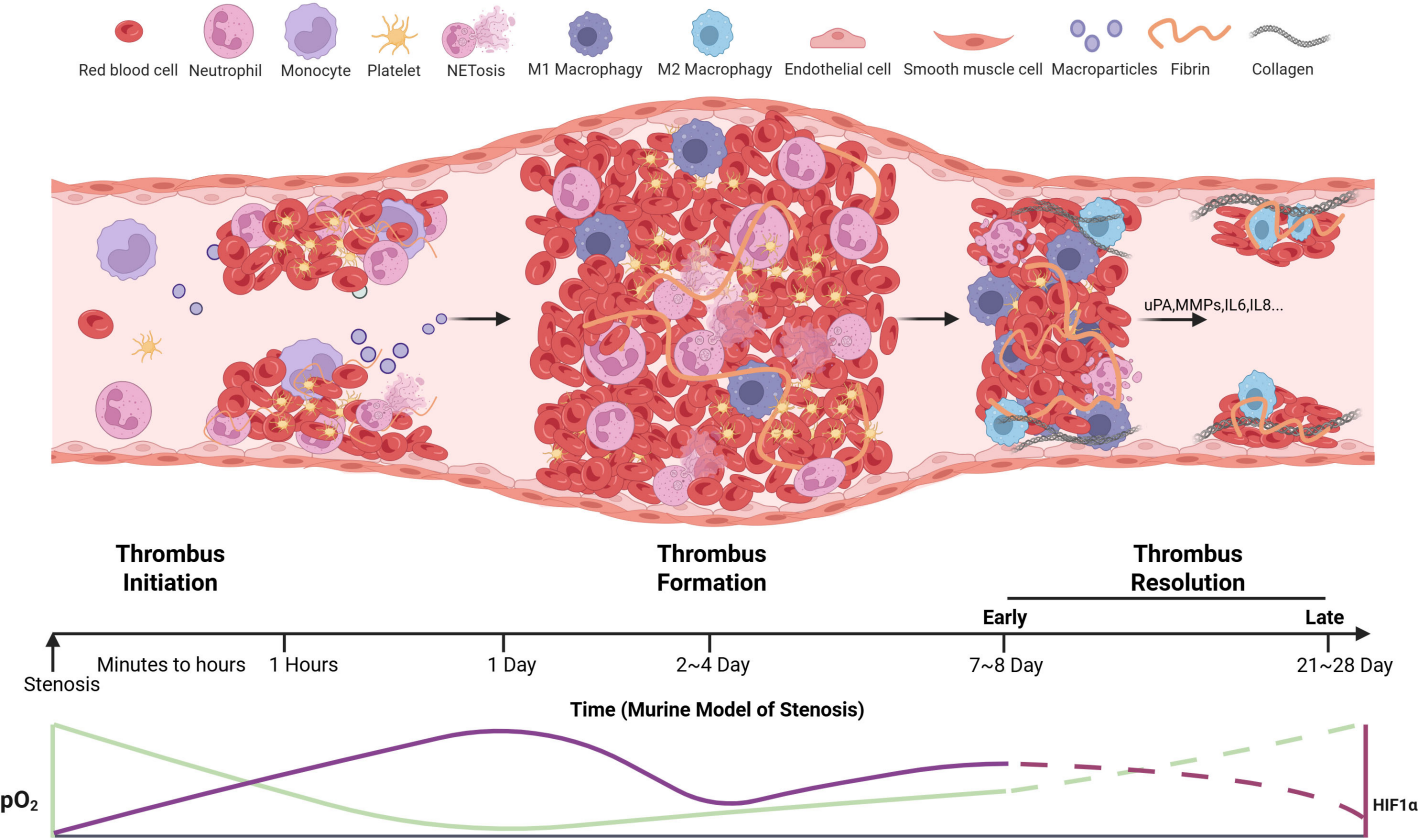
Natural evolution of venous thrombosis and the associated changes in pO_2_ and HIF1α. This diagram integrates the temporal progression of venous thrombosis with the dynamic immune response. The timeline, based on murine stenosis models, delineates three stages of thrombus development: initiation (minutes to hours), formation (peaking at 2–4 days), and resolution (beginning at 7–8 days, with complete recanalization by 21–28 days). The top panel illustrates the sequential infiltration and dominant roles of key immune cells: activated platelets and neutrophils drive initiation; neutrophil extracellular traps (NETs) and monocytes dominate the growth and stabilization phase; and M1 and M2 macrophages facilitate resolution and remodeling. The graphs depict the inverse relationship between intraluminal pO_2_ (markedly decreased by day 1) and HIF-1α expression throughout thrombus evolution. The dotted portion of the HIF-1α curve represents a hypothesized trajectory that requires further empirical validation.

The natural evolutionary process of experimental VT includes initiation, formation and resolution (**Figure 1**). In the presence of external stimuli, such as hypoxia, VT is initiated in a short time (minutes to hours) (14) through a series of activation events involving endothelial cells, platelets, neutrophils, monocytes, erythrocytes and coagulation pathways (15, 16). Thrombus formation begins at 1 hour after stenosis, and a fragile thrombus is formed at 1 day (17). The thrombus grows into a hard thrombus within 2~4 days when the weight of the thrombus reaches its maximum and the initial thrombus is replaced by a mat of medium-textured fibrin (17, 18). Thrombus formation is accompanied by its resolution, making it difficult to clearly separate these two phases. Generally, thrombus resolution occurs 7~8 days post-thrombosis when fibrinolysis occurs at a high rate, collagens appear and thrombus-associated immune cells are induced to produce inflammatory cytokines and various proteases (19). In the late phase of thrombus resolution, which occurs 14~21 days post-thrombosis or even longer, the fibrosed thrombus retracts into the vessel wall, which results in vascular recanalization (20). The natural progression from thrombosis to thrombus resolution generally takes 21–28 days in murine models and approximately 1 year in humans (18, 21).

## 3 Hypoxic microenvironment

**Figure 2:**
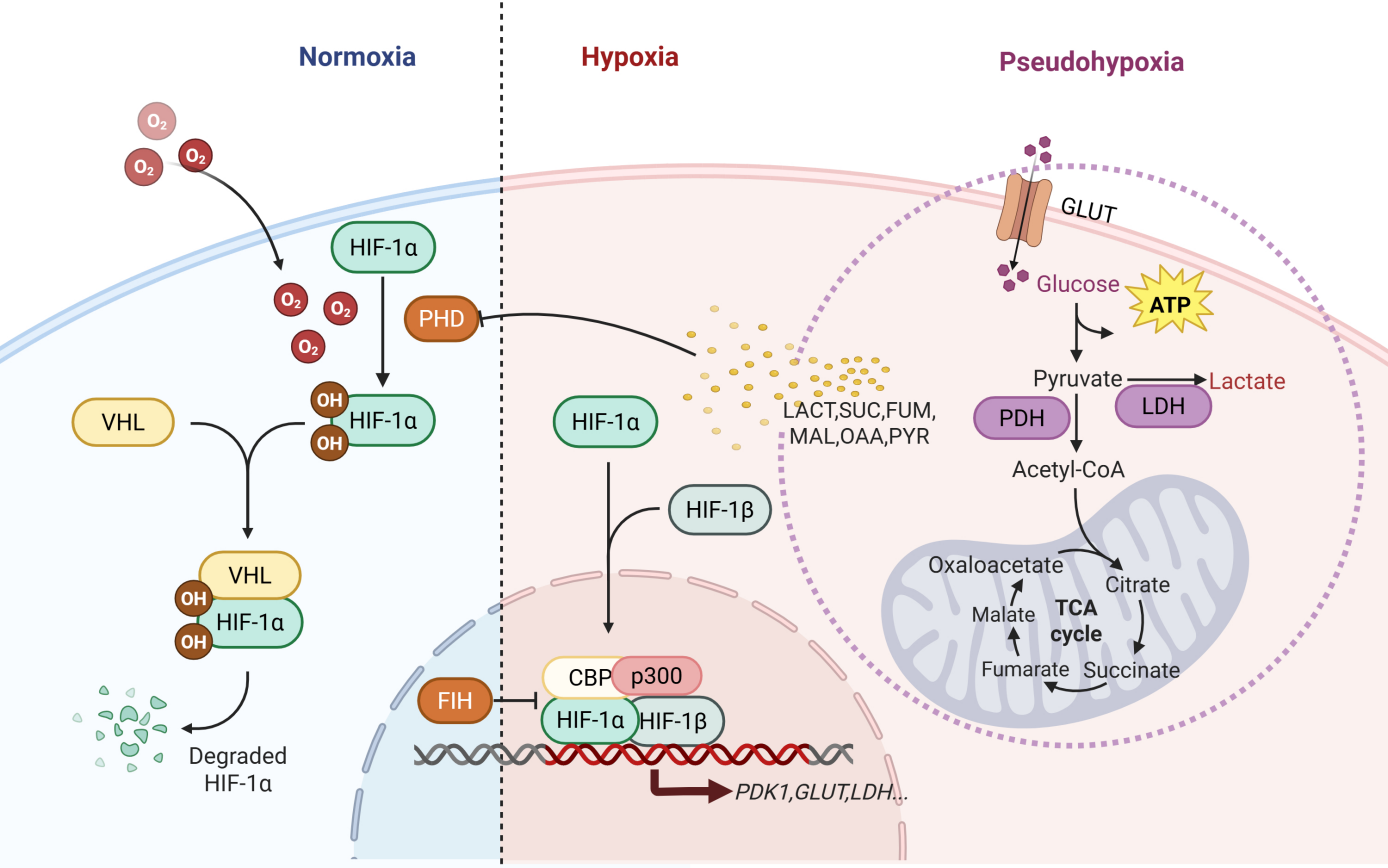
Molecular mechanisms of HIF-1α regulation and metabolic reprogramming under hypoxia and pseudohypoxia. The left panel illustrates HIF-1α regulation. Under normoxia, HIF-1α is hydroxylated by prolyl hydroxylases (PHDs), leading to VHL-mediated proteasomal degradation. Additionally, factor inhibiting HIF (FIH) hydroxylates HIF-1α under normoxia, blocking CBP/p300 recruitment and thereby suppressing transcriptional activity. Under hypoxia, PHD activity is inhibited, allowing HIF-1α to stabilize, translocate to the nucleus, dimerize with HIF-1β, and recruit transcriptional coactivators such as CBP/p300.The right panel depicts downstream metabolic changes induced by HIF-1α, including upregulation of glucose transporters (GLUT), enhanced glycolysis (glucose → pyruvate → lactate), inhibition of pyruvate dehydrogenase (PDH), activation of PDK1 and LDHA, and an altered TCA cycle with accumulation of succinate, fumarate, and other intermediates (malate, oxaloacetate, citrate). These metabolites cause product inhibition of PHD, leading to HIF-1α stabilization and activation even under normoxic conditions—a phenomenon termed pseudohypoxia (indicated within the purple dashed line).

Adaptation to low oxygen tension (hypoxia) in cells and tissues leads to the transcriptional induction of a series of genes that participate in angiogenesis, glucose metabolism, matrix metabolism and cell proliferation/survival (22). The primary factor mediating this response is hypoxia-inducible factor-1 (HIF-1), an oxygen-sensitive transcriptional activator (23). HIF-1 is a heterodimer composed of a subunit of HIF-1β, which is constitutively expressed, and a subunit of HIF-1α (or its paralogues HIF-2α and HIF-3α), the expression and transcriptional activity of which are precisely regulated by the cellular O_2_ concentration (22). In the presence of oxygen (normoxia), HIFα interacts with and binds to the von Hippel–Lindau (VHL) protein, which consequently activates the ubiquitin ligase system, leading to proteasomal degradation of HIFα. Hydroxylation of proline residues in HIFα is vital for VHL binding and depends on a-ketoglutarate-dependent dioxygenases, prolyl hydroxylases (PHDs), and the asparaginyl hydroxylase factor-inhibiting HIF (FIH) (24). During hypoxia, PHDs and FIH are inactive, leading to HIF-1α stabilization and dimerization with HIF-1β. Consequently, HIF1 binds with the coactivator cyclic AMP response element-binding protein (CBP)/p300, promoting the expression of HIF target genes (25) (**Figure 2**).

The effect of hypoxia can be enhanced by pseudohypoxia (26–29), a phenomenon caused by cell metabolic changes after hypoxia. Individual cells must adapt to O_2_ deprivation by reprogramming their metabolism, shifting their primary metabolic strategy from predominantly mitochondrial respiration towards increased glycolysis to maintain ATP levels. Hypoxia, via HIF-1-mediated activation of the pyruvate dehydrogenase kinase 1 (PDK1) gene (30), triggers increased glycolysis and decreased respiration: PDK1 phosphorylates the catalytic subunit of pyruvate dehydrogenase (PDH), the enzyme that converts pyruvate to acetyl coenzyme A (AcCoA) for entry into the mitochondrial tricarboxylic acid (TCA) cycle, thereby generating reducing equivalents for the electron transport chain. The reduced delivery of substrate to the mitochondria for oxidative phosphorylation results in reduced ATP synthesis, which must be compensated for by increased glucose uptake via glucose transporters (GLUT1, GLUT3) and increased conversion of glucose to lactate by the activity of glycolytic enzymes and lactate dehydrogenase A (LDHA), which are all encoded by HIF-1 target genes (31). In pseudohypoxia, accumulation of metabolic intermediates from glycolysis and the citric acid cycle — including succinate, fumarate, oxaloacetate, pyruvate, lactate, and malate — causes product inhibition of PHD, resulting in HIF-1α stabilization and activation (**Figure 2**).

## 4 Hypoxic microenvironment in the initiation and formation of VT

**Figure 3:**
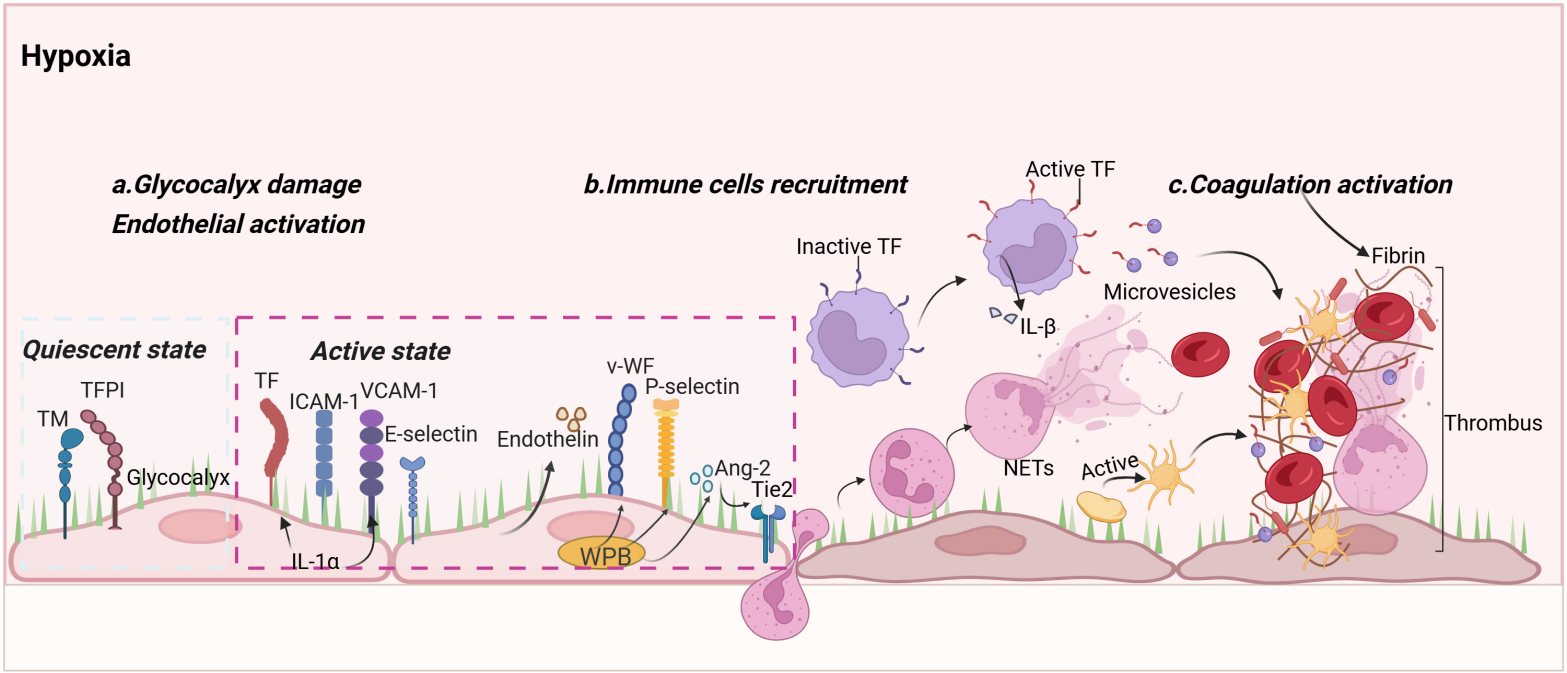
Hypoxia-induced endothelial activation, immune cell recruitment, and coagulation activation. Under hypoxic conditions, endothelial glycocalyx damage occurs, leading to endothelial activation. The anticoagulant molecules, such as tissue factor pathway inhibitor (TFPI) and thrombomodulin (TM), are downregulated as the endothelium shifts from a quiescent to an active state. This is accompanied by elevated levels of inflammatory mediators (e.g., IL-1α), upregulation of adhesion molecules (ICAM-1, VCAM-1, E-selectin), increased expression of tissue factor (TF) and Weibel-Palade body (WPB) contents (including von Willebrand factor [vWF] and P-selectin), as well as alterations in Ang-2/Tie2 signaling. These events promote immune cell recruitment, release of microvesicles and neutrophil extracellular traps (NETs), and ultimately activate the coagulation system, leading to fibrin deposition and thrombus formation.

The first experimental evidence that thrombogenesis is triggered by hypoxia was provided by Hamer et al., who measured the oxygen tension in the valvular sinus of 8 dogs and 2 patients during streamline blood flow and in conditions of intermittent pulsatile flow (7). The blood within the valve pockets became rapidly hypoxic when undisturbed during streamline flow, and a steeply declining pO_2_ gradient from approximately 5 kPa to 0.1 kPa was observed from the top to the bottom of the sinus after 2 hours of stasis. The anatomical location of severe, stasis-associated hypoxia—the deepest recess of the sinus—is the same as that of thrombus initiation (32). Hypoxia in the deep region of venous valves induces an adaptive response within local endothelial cells and blood cells through several oxygen-sensing mechanisms, which can cause thrombosis if this response is disrupted (**Figure 3**).

### 4.1 Endothelial cells

Endothelial cells (ECs) that lie on the inner surface of blood vessels and contact blood directly are called vascular endothelial cells (VECs) and play critical roles in the control of vascular function. When the vessel wall is breached or the endothelium is disrupted, collagen and tissue factors become exposed to the flowing blood, thereby activating the coagulation pathway and initiating the formation of a thrombus (33). In contrast to arterial thrombosis, endothelial cells participate in venous thrombosis through functional changes rather than denudation or injury (32, 34).

The first endothelial target of hypoxia is the endothelial glycocalyx (35), which is a gel-like layer comprised of membrane-attached proteoglycans, glycosaminoglycan chains, glycoproteins and adherent plasma proteins that covers the luminal surface of vascular endothelial cells (36) (**Figure 3**) and has anticoagulant and antiadhesive effects. Hypoxia causes damage to the glycocalyx, resulting in coagulation activation by thrombin formation and the binding of platelets and neutrophils (37).

Second, hypoxia strongly changes the cell factors secreted by ECs, leading to the adherence of cellular components. In detail, hypoxia-induced exocytosis of the EC Weibel-Palade (WP) body increases vWF (38–40) and P-selectin (39, 41), which promote platelet and leukocyte adhesion, respectively. The release of vWF is also promoted by increased endothelin (42, 43). Hypoxia also causes energy deficiency in endothelial cells, leading to (Ca^2+^)-induced stimulation of ECs and subsequent activation of phospholipase A2 (PLA2) to increase lyso-PAF release, which ultimately transforms into the neutrophil adhesion molecule PAF (44, 45). The other factors associated with increased neutrophil adherence included ICAM-1, E-selectin (45–47) and VCAM-1 (summarized in **Table 1**; **Figure 3**).

**Table 1:** The immune cells change in hypoxic microenvironment during venous thrombus initiation, formation and resolution.

Cell types	Targets		Key immune mechanism	Changes after hypoxia	Effect on thrombus	References
Endothelial cell	glycocalyx		1. A barrier opposing vascular permeability 2. Anti-coagulant and anti-adhesive effect on the surface of ECs	Damage Coagulation activation Binding of platelets and neutrophils	Initiation Formation	(35, 36)
	Cell factors	vWF	1. Coagulation (VIII carrier) 2. Platelet adhesion 3. Slow blood flow	Exocytosis of endothelial cell Weibel-Palade Bodies	Initiation	(38–40)
		P-selectin	Leukocyte adhesion	Exocytosis of endothelial cell Weibel-Palade bodies	Initiation	(39, 41)
		TF	A key initiator of the extrinsic coagulation cascade	↑	Initiation	(48, 49)
		ICAM-1, E-selectin, VCAM-1, PAF	Neutrophils adherence	↑	Initiation Formation	(44–47)
		Factor X activator	Promote coagulation	↑	Initiation Formation	(49–52)
		Endothelin	1.Release of tPA and vWF 2.Enhance tissue factor and suppress thrombomodulin expression	↑	Initiation Formation	(42, 43)
		TM	Anticoagulation	Membrane TM/soluble TM↓	Formation	(53, 54)
		TFPI	The endogenous inhibitor of the TF	↓	Formation	(55)
		IL-1α	Mediate TF and VCAM gene expression	↑	Formation	(56)
		VEGF, Ang-4	1. Maintain vascular homeostasis 2. Extracellular matrix invasion and tube formation	↑	Resolution	(57)
	EndMT		Lost anticoagulation and thrombolytic function	Occur	Formation	(58)
Neutrophil	NETs		1. Trigger the coagulation pathway and platelet aggregation 2. Provide a scaffold to recruit red blood cells, platelets and leukocytes	Promote	Formation	(59)
	HIF1 targets		1. Ang 1, matrix degradation 2. Endouthelin 1, vascular permeability 3. MMPs, degrade collagen 4. VEGF, angiogenic effect	Occur	Resolution	(12)
Monocyte	Macro-particles	TF	A key initiator of the extrinsic coagulation cascade	↑	Initiation Formation	(60)
Platelet	Cytosolic Ca^2+^		1. Initiate an acute inflammatory response 2. Provide a surface for coagulation	Activated	Formation	(61)
Erythrocyte	Red cell mass		Erythropoiesis	Gene mutation induced pseudohypoxia	Initiation Formation	(62)
Macrophage	M1 macrophages		Activate NLRP3 inflammasome complex and produce IL-1β	Stimulate	Formation	(63)
	M2 macrophages		Collagen turnover and degradation	Occur	Resolution	(64)

↑, increase; ↓, decrease.

Third, hypoxia influences the vascular coagulation state. According to the cell-based model, coagulation is regulated by the properties of cell surfaces (65). Hypoxia upregulates tissue factor (TF) expression on venous endothelial cells (48–50). As the primary initiator, TF binds FVII/FVIIa to activate both FX and FIX, producing trace amounts of thrombin. This initial thrombin then triggers an amplification loop that activates platelets and cofactors (FV, FVIII, FXI) on the platelet surface, thereby setting the stage for large-scale thrombin generation during the propagation phase. In parallel, hypoxia lowers the level of tissue factor pathway inhibitor (TFPI) (55), the key inhibitor of TF-triggered coagulation. Hypoxia also diminishes the thrombomodulin (TM) antigen, attenuates its functional activity on the cell surface by 80-90% and suppresses its mRNA (53) through an epigenetic mechanism (66). TM is an anticoagulant that binds to thrombin and then deactivates it to initiate an anticoagulation cascade (67). The thrombin-TM complex activates protein C, which is a crucial mediator that inactivates the coagulant factors Va and VIIIa and further reduces thrombin generation in anticoagulation processes.

Finally, hypoxia enhances thrombosis via endothelial-mesenchymal transition (EndMT). EndMT is a process in which ECs lose their endothelial specification and acquire mesenchymal cell features. It is progressively implicated in the development and progression of pulmonary hypertension (PH) and pulmonary arterial hypertension (PAH) in response to hypoxia (68). EndMT begins with a morphological change from a classical cobblestone-like structure to an elongated, spindle shape. The process further involves a loss of cell–cell adhesion, dissociation from the basement membrane, and migration into the medial layer. Studies on iliac vein compression have indicated that EndMT may enhance thrombosis through the TGF-β/Smad3 signaling pathway (58, 69).

### 4.2 Neutrophils and monocytes

At the initial stage of thrombus formation, neutrophils were the predominant leukocyte subset within venous thrombi, constituting approximately 70% of all accumulated leukocytes, whereas monocytes accounted for about 30% (15). Under normal conditions, neutrophils and monocytes do not adhere to the endothelium. However, in ligature-based infrarenal vena cava (IVC) mouse models, it has been demonstrated previously that the vein endothelium has an inflammatory effect, as it can recruit leukocytes to the vein wall via cell adhesion molecules (CAMs) (15). Hypoxia, on the other hand, leads to sterile inflammation, and conversely, inflammatory responses alter the endothelial structure and increase endothelial permeability in the IVC, promoting venous thrombosis. Upon activation, neutrophils can release nuclear material known as NETs, as demonstrated in a murine model of DVT induced by stenosis in the IVC; this process was initially described as a part of antimicrobial defence. The prothrombotic effect of NETs involves triggering the coagulation pathway and platelet aggregation. NETs provide a scaffold to recruit red blood cells, platelets and leukocytes, as well as to bind plasma proteins, such as VWF and fibronectin. This scaffold also supports fibrin deposition by binding with fibrinogen. In addition, two primary components of the NET structure, DNA and histones, are involved in coagulation pathways, which subsequently contribute to further thrombus development (70). Ribonucleoprotein immune complexes (RNP ICs), inducers of NETosis, require mitochondrial reactive oxygen species (ROS) for maximal NET stimulation (71). Hypoxia potentiates the release of ROS. Recently, Joseph A. et al. showed that neutrophils can use their mitochondria to generate ROS and that mitochondrial ROS release is increased under hypoxic conditions. Significantly, neutrophils stabilize HIF-1α through the release of mitochondrial ROS via the glycerol 3-phosphate shuttle (59).

Clinical studies have confirmed that high levels of circulating extracellular vesicles (EVs) may represent an independent risk factor for VTE (72). Furthermore, an experimental study in humans showed that acute 12% O_2_ exercise increases the release of FV/FVIII-rich, phosphatidylserine (PS)-exposed, and TF-expressing microparticles from monocytes, thereby enhancing susceptibility to monocyte-related thrombosis under hypoxia (73). Supporting these clinical observations, a murine model of whole-animal hypoxia demonstrated that monocytes and monocyte-derived TF-positive EVs promote thrombosis, and that hypoxia-induced mononuclear phagocyte recruitment and TF expression, mediated via the TLR3-ERK1/2-AP1 pathway, trigger local fibrin deposition (60).

Hypoxia can stimulate monocytes/macrophages (Mo/MΦs) via the hypoxia–HIF-1α–NLRP3–IL-1β axis to produce IL-1β, which potentiates venous thrombosis in response to hypoxia (63). It is well established that monocyte activation and monocyte-derived microvesicles (MVs) contribute to thrombosis under hypoxic conditions. However, during the initiation phase of thrombogenesis, their role appears to be less critical than that of neutrophils. For instance, monocytes typically constitute only a modest fraction (approximately 20–30%, depending on the model and time point) of recruited leukocytes in early venous thrombi (15). Moreover, a recent study demonstrated that monocytes/macrophages (Mo/MΦs) are not essential for thrombogenesis: in murine models of stasis and stenotic IVC, an 80–90% reduction in circulating CD11b^+^ Mo/MΦs did not significantly affect thrombus size, neutrophil recruitment, or NET formation, suggesting that neutrophils can drive thrombus formation largely independently of monocytes in these settings (74). Although the full contributions of monocytes continue to be elucidated, their best-defined roles in coagulation are to initiate coagulation through TF presentation and to potentiate thrombo-inflammation via inflammasome activation (75).

### 4.3 Platelets and erythrocytes

It has long been believed that platelets play a unique role in the process of thrombosis because their participation is crucial in arterial thrombosis but not pronounced in venous thrombosis. A candidate mechanism for platelet recruitment is adhesion to VWF via VWF-platelet GPIb interactions (76). Alexander et al. demonstrated in a stenosis model of DVT that VWF-/- mice are completely protected from venous thrombosis, although P-selectin and/or other constituents of WPBs could be independently involved (38). This experimental result also confirmed that platelet recruitment by VWF appears pivotal for subsequent thrombus formation. Activated platelets in the circulation can initiate an acute inflammatory response by inducing P-selectin expression on the endothelium and consequently stimulating leukocyte rolling on the vessel wall. Simultaneously, activated platelets provide a surface for coagulation factor assembly and TF recruitment, which may be important contributors to DVT initiation and development. A recent study in which rats were exposed to acute stimulated hypoxia of 8% oxygen suggested that a hypoxic environment alters the platelet proteome and induces platelet hyperreactivity, leading to a prothrombotic phenotype that is mainly mediated by the activation of calpains, which are activated by elevated cytosolic Ca^2+^ (61). The coagulation or platelet function of healthy men exposed to acute hypoxia (8% O_2_) for a short time did not significantly change; however, healthy human subjects continuously exposed to prolonged hypobaric hypoxia due to high altitude environmental exposure for 2 months (3,700 m, which is equivalent to ~12,139 feet above sea level) exhibited significant platelet activation, as demonstrated by increased engagement and adhesion to fibrinogen, fewer alpha granules by transmission electron microscopy, increased circulating PF4 and ADP, and significantly enhanced clot retraction (77). In general, hypoxia can directly promote platelet activation or indirectly activate platelets through prothrombotic factors, thereby promoting the occurrence and development of thrombosis.

It is widely known that red blood cell numbers and haemoglobin concentrations increase during altitude acclimatization. Hypobaric hypoxia is associated with an increase in erythropoiesis and an increased thrombotic risk. Hypoxia stimulates erythropoietin production by the kidney to increase the red blood cell mass via the HIF1 pathway (62). Chuvash polycythaemia (CP) is a rare congenital form of polycythaemia caused by homozygous R200W and H191D mutations in the VHL gene, whose gene product is the principal negative regulator of HIFα (78). The impairment of HIF-α degradation leads to increased transcription of many HIF-regulated genes, including erythropoietin, and to elevated haematocrit levels and thrombosis. Deregulation of the HIF pathway contributes to the elevated rate of thrombosis in patients and mice with CP. Germline mutations in genes such as EGLN1 (encoding PHD2), EPAS1 (encoding HIF2α), and the EPO receptor can also result in altered erythropoiesis, such as EGLN1950 C>G, EGLN11112 G>A, EPAS11609 G>T and EPAS11604 T>C (16).

## 5 Hypoxic microenvironment in venous thrombus resolution

Venous thrombus resolution is a complex process resembling sterile wound healing and is achieved through the synergy of four key mechanisms, namely, inflammation, angiogenesis, fibrinolysis and collagenolysis. Hypoxia is characteristic of many diseased tissues (such as solid tumors and atherosclerotic plaques), and occlusive venous thrombi are also hypoxic, as demonstrated by the Evans team (11–13). Intraoperatively, they introduced a state-of-the-art oxygen microsensor that directly quantifies the real-time oxygenation of mouse thrombi through the vein wall to show that oxygen levels within newly formed venous thrombi are reduced by 90% compared with those in venous blood (13). HIF1α expression is negatively correlated with pO_2_. Using a mouse model of venous thrombosis, Evans et al. assessed HIF1α levels during natural thrombus resolution from day 1 to day 7. Since the thrombus had become too small to permit HIF1α measurement by day 14 (13), we provide a predictive estimate in **Figure 1**. At present, studies on the involvement of hypoxia in thrombus resolution have focused on the upregulation of HIF1α. HIF1-mediated angiogenic factors such as vascular endothelial growth factor (VEGF), angiopoietin 1 (Ang-1), endoglin, endothelin 1, IGFBP1, leptin, MCP1, MMP9, PDGF-B, PLGF, SDFI and TIMPI are increased, enhancing thrombus resolution (11, 13, 79). A hypoxia-induced increase in glycolytic metabolites is also a major feature of thrombosis. Glycolytic metabolites affect the degradation of HIF1α and are also involved in protein posttranscriptional translational modification (PTM). It is likely that hypoxia induces an increase in glycolytic metabolites to play a role in the resolution of thrombi. Venous thrombi resolve naturally through a slow process of organization and recanalization. Accelerating this process may help prevent PTS by reducing valvular damage and residual obstruction, and would also be valuable when thrombolysis is contraindicated.

### 5.1 Endothelial cells and angiogenesis

HIF-1 activates the transcription of genes encoding angiogenic growth factors, which are secreted by macrophages and stimulate endothelial cells, leading to angiogenesis (80). However, HIF-1 plays an equally profound role as a mediator of EC-autonomous responses to hypoxia and increases the mRNA and protein levels of VEGF, angiopoietin-2 (Ang-2), and angiopoietin-4 (Ang-4), as manifested by extracellular matrix invasion and tube formation (57). VEGF is an endothelial cell–specific mitogen that plays an important role in vascular development and angiogenesis during embryogenesis, wound healing, solid tumor growth, and certain chronic fibroproliferative inflammatory diseases. Autocrine VEGF, which is released by endothelial cells, maintains vascular homeostasis and simultaneously serves to amplify the angiogenic effects of VEGF (79, 81). VEGF and IL-8 are secreted by endothelial cells, thereby enhancing endothelial cell migration and angiogenesis via Src/Vav2/Rac1/PAK1 signaling (82). Eight hours after the onset of hypoxia, a large number of HIF-1 regulatory genes encoding cytokines/growth factors, receptor tyrosine kinases, G protein-coupled receptors, and related signaling proteins provide a broad molecular basis for EC activation. In addition to autonomous cellular effects, hypoxia-induced gene products in ECs have more global implications for vascular biology, such as prostaglandins and genes encoding collagens and collagen-modifying enzymes (83).

Angiogenesis and an intact angiogenic response following acute thrombosis or thromboembolism are key mediators of normal thrombus resolution. Conversely, incomplete resolution of thrombus material accompanied by fibrotic remodeling impairs venous thrombus resolution, potentially leading to persistent venous obstruction and thrombus extension (84). The angiopoietins—Ang-1 and Ang-2—are growth factors critical for vascular development. Ang-1 promotes vessel maturation, adhesion, migration, and endothelial cell survival, whereas Ang-2 disrupts vascularization and promotes endothelial cell death. In patients with VTE, serum Ang-1 levels are significantly decreased while Ang-2 levels are increased, reflecting endothelial dysfunction and vascular instability (85). Mechanistically, Ang-1 constitutively activates its receptor Tie2, thereby promoting vascular stability and endothelial survival. In contrast, Ang-2 acts as a context-dependent antagonist: it competitively binds to Tie2 but fails to induce sufficient receptor phosphorylation, thus blocking the protective signals of Ang-1. Under hypoxic conditions—common in venous thrombi—Ang-2 is rapidly released from endothelial Weibel-Palade bodies (86). Gain-of-function experiments in mice following IVC ligation demonstrated that exogenous Ang-2 administration and transgenic endothelial Ang-2 overexpression delayed VT resolution, with thrombi showing lower Tie2 phosphorylation and fewer microvessels (87). Thus, hypoxia-mediated Ang-2 overexpression shifts the Ang-1/Ang-2 balance toward Tie2 inhibition, leading to impaired microvessel formation and delayed thrombus resolution.

Endothelial progenitor cells (EPCs), which are multipotent progenitor cells that reside mainly in human bone marrow, have emerged as a promising therapeutic option for DVT-related thrombus resolution. Circulating endothelial and pericyte progenitors that express the stromal-derived factor 1 (SDF-1) receptor CXCR4 are recruited to and retained in ischaemic tissues through HIF-1α-mediated upregulation of the chemoattractant cue SDF-1α at these sites. In addition, the overexpression of VEGF, a target of HIF-1α, stimulates angiogenesis by upregulating SDF-1 and thereby recruits CXCR4-positive proangiogenic myeloid cells. HIF-1α also induces the expression of the intracellular adhesion molecule ICAM-1, which serves as a docking site for mobilized EPCs in ischaemic tissues (88).

### 5.2 Neutrophils, macrophages and macrophage polarization

In the early stages of venous thrombus resolution, neutrophils are the predominant leukocyte subset, promoting fibrinolysis and collagenolysis. The formation and resolution of venous thrombi occur simultaneously. Neutropenia impairs DVT resolution in a rat model of venous thrombosis, resulting in significantly larger thrombi (89). Neutrophils are the predominant nucleated cell type present in newly formed and 1-day-old thrombi (90). Decreased oxygen initiates HIF stabilization in neutrophils, and HIF activity promotes phagocytosis and inhibits apoptosis to increase the phagocyte lifespan (91). Later in the process of thrombus remodeling, neutrophils are replaced by monocyte-derived macrophages, the predominant cell type in the thrombus. Monocytes migrate into the thrombus during natural resolution, possibly via the vein wall, and convert to the macrophage phenotype in the thrombus. Morphometrically, macrophages start appearing in the thrombus on Day 1 and culminate around Day 7 (90). Macrophages are widely known as important elements of innate immunity; however, they can secrete growth factors and cytokines, as well as proteolytic enzymes, to remodel the extracellular matrix, and in doing so, they play crucial roles in angiogenesis and homeostasis. Injection of peritoneal macrophages can decrease the experimental thrombus size by 5-fold, whereas monocyte chemotactic protein-1 (MCP-1, also known as CCL2) can decreased the thrombus size by 6-fold (92, 93). Tissue macrophages can exist in two different activation states: the proinflammatory classically activated pathway, known as the M1 pathway, and the anti-inflammatory alternatively activated pathway, known as the M2 pathway. Whereas M1-polarized macrophages are associated with the initial stage of tissue injury and secrete cytokines and other factors that amplify the inflammatory response, M2-polarized macrophages are generally involved in the tissue clearing and dissipation of inflammation. Both M1 and M2 macrophages are present in venous thrombosis (VT), and functional M2 macrophages—characterized by high expression of CD206, Arg1, and FIZZ1, as well as upregulation of VEGF, MMP-9, uPA, and other molecules—serve as the core drivers of thrombus resolution. They promote resolution through the following mechanisms: secreting VEGF, basic fibroblast growth factor (bFGF), and other factors to enhance neovascularization (recanalization); releasing MMP-2, MMP-9, uPA, and related enzymes to degrade fibrin and collagen; producing anti-inflammatory cytokines such as IL-10 to restrain excessive inflammation; and clearing necrotic cells and NETs via phagocytosis (64, 94–96).

Epigenetic modifications have emerged as critical determinants of macrophage plasticity and heterogeneity. Accumulating evidence indicates that lactate-induced changes in histone lactylation levels within macrophages can modulate gene transcription (97). In response to hypoxia, cells shift their metabolism from oxidative phosphorylation to glycolysis, leading to increased lactate production, which then serves as a precursor for histone lactylation (98). In hypoxia-challenged M1 macrophages, the endogenous “lactate clock” and “intracellular lactate shuttle” promote the expression of genes associated with homeostasis, some of which—such as *vegfa*—are linked to an M2−like phenotype and are not regulated by HIF1α during M1 polarization. During the early phase of myocardial infarction (MI), elevated lactate levels in macrophages enhance histone lactylation, thereby inducing the transcription of Lrg1, VEGF−a, and IL−10. Moreover, high lactate concentrations in cells with fragmented mitochondria further promote histone lactylation, facilitating the transition of macrophages toward an anti−inflammatory phenotype (99). This mechanistic insight into histone lactylation not only deepens our understanding of disease pathogenesis but also opens new avenues for therapeutic intervention.

## 6 Conclusion

Whether high-altitude hypoxia is an independent risk factor for VT remains controversial, as its effects are modulated by multiple variables, including altitude, exposure duration, thrombophilia, and immobilization (9, 100, 101). To resolve this controversy, it is essential to distinguish between acute and chronic hypoxic responses during VT. Acute hypoxia rapidly alters immune cell metabolism and activity, which may promote thrombus formation. In contrast, chronic hypoxia induces long−lasting adaptations in immune responses via oxygen−guided regulation of signaling cascades and epitranscriptomic programs, leading to gene expression changes that drive thrombus resolution and vascular remodeling (102).

A large body of *in vivo* and *in vitro* evidence has confirmed that hypoxia is indeed an independent risk factor for VT—not only external environmental hypoxia (e.g., high altitude) but also internal environmental hypoxia resulting from diseases such as COVID-19, sepsis, obstructive sleep apnoea (OSA), and chronic obstructive pulmonary disease (COPD) (4, 103–107). Hypoxia promotes thrombosis and also influences thrombus resolution. In general, acute hypoxic responses involve alterations in pre−existing protein activities that initiate thrombosis, whereas chronic hypoxic responses involve gene expression changes that primarily affect thrombus resolution. The response of endothelial cells to changes in oxygen availability critically depends on the duration of exposure: in the acute phase, endothelial cells release inflammatory mediators to promote immune cell recruitment and adhesion; under chronic hypoxia, a battery of transcription factors (e.g., HIF−1α) is activated, potently inducing growth factors and cytokines such as PDGF and VEGF (108).

During VT evolution, neutrophils are gradually replaced by monocyte−derived macrophages, highlighting the important role of macrophages in thrombus remodeling. However, the relationships among hypoxia, macrophage polarization, and thrombus resolution remain incompletely understood and warrant further investigation. Notably, increased glycolytic metabolites under hypoxia not only create a pseudohypoxic state that inhibits HIF−1α degradation but also may affect macrophage polarization through protein post−translational modifications (PTMs). Both mechanisms may positively influence venous thrombus resolution. A comprehensive understanding of the entire evolution of venous thrombosis (initiation, formation, and resolution) and identification of key modulating factors are of great importance for the prevention and treatment of VT and for reducing the incidence of PTS.
